# Daily Intake of Paraprobiotic *Lactobacillus amylovorus* CP1563 Improves Pre-Obese Conditions and Affects the Gut Microbial Community in Healthy Pre-Obese Subjects: A Double-Blind, Randomized, Placebo-Controlled Study

**DOI:** 10.3390/microorganisms8020304

**Published:** 2020-02-22

**Authors:** Tomonori Sugawara, Daisuke Sawada, Sae Yanagihara, Yumeko Aoki, Isao Takehara, Hirosuke Sugahara, Tatsuhiko Hirota, Yasunori Nakamura, Susumu Ishikawa

**Affiliations:** 1 Core Technology Laboratories, Asahi Quality and Innovations, Ltd., 11-10, 5 Chome, Fuchinobe, Chuo-ku, Sagamihara-shi, Kanagawa 252-0206, Japan; 2 PI-Food Service Division, Clinical Support Corporation, 4-1, Nishi 8 Chome, Minami 1 jo, Chuo-ku, Sapporo-shi, Hokkaido 060-0061, Japan; 3 Medical Corporation Shoureikan Sinsapporo Seiryo Hospital, 1-30, 2 Chome, Higashi 4 jo, Atsubetsu, Atsubetsu-ku, Sapporo-shi, Hokkaido 004-0004, Japan

**Keywords:** *Lactobacillus amylovorus* CP1563, paraprobiotics, 10-hydroxyoctadecanoic acid, obesity, clinical trial, gut microbiota, butyrate-producing bacteria

## Abstract

Despite the fact that gut microbiota is closely associated with obesity, few studies have focused on the influences of paraprobiotics as food ingredients on both obesity prevention and the gut microbial community. In this study, we evaluated the effects of fragmented *Lactobacillus amylovorus* CP1563 (CP1563) as a paraprobiotic for obesity prevention and investigated its effects on the gut microbial community in pre-obese subjects. One hundred sixty-nine healthy subjects with a body mass index from 25.0 to 29.9 kg/m^2^ ingested beverages with or without the fragmented CP1563 containing 10-hydroxyoctadecanoic acid (10-HOA) for 12 weeks. The changes in abdominal, total, visceral, and subcutaneous fatty areas were significantly lower in the CP1563-10-HOA group than in the placebo group at 12 weeks. Furthermore, 16S rRNA gene sequencing of fecal DNA revealed that the changes in the abundances of the genera *Roseburia* and *Lachnospiraceae;*g were significantly greater in the CP1563-10-HOA group than in the placebo group, and the changes in the abundances of the genus *Collinsella* was significantly smaller in the CP1563-10HOA group than in the placebo group. Our results showed that continuous ingestion of the fragmented CP1563 containing 10-HOA reduced abdominal body fat and affected the gut microbial community in pre-obese healthy subjects. Our findings may contribute to the understanding of the relationship between the anti-obesity effect of paraprobiotics and gut microbiota.

## 1. Introduction

Obesity causes lifestyle-related metabolic disorders, including hypertension, dyslipidemia, and diabetes, which lead to advanced atherosclerosis or cardiovascular diseases [1,2]. The development of obesity is caused by genetic susceptibility, fitness habits, and dietary habits. In particular, the daily energy imbalance gap between intake and expenditure has a major impact on weight gain. Because the risk of weight gain increases with the excess energy intake characteristic of Western eating habits [3], the energy imbalance gap is considered to be closely associated with the Westernization of eating habits. In addition, eating habits are known to modulate the gut microbial community [4], and the gut microbiota plays an important role in host health maintenance and disease pathogenesis [5]. A previous report related to obesity showed that a Western diet containing high fat and high sugar altered the gut microbial community [6,7]. In addition, in a microbiota transplantation study using germ-free mice, the mice with transplants from donor mice fed a Western diet experienced significant weight gain compared to the other mice with transplants from donor mice fed a non-Western diet [6]. Another study showed that a gut microbiome profile associated with obesity influenced intestinal permeability [8] and inflammation status [9,10], and these changes were associated with the induction of obesity. Given these insights, both dietary habits and gut microbial conditions need to be taken into account in obesity prevention.

Many studies have shown beneficial effects with regard to obesity prevention induced by the consumption of certain food ingredients, which is a strategy related to dietary habits [11,12,13]. In addition, probiotics, which constitute a food ingredient, are valuable for obesity prevention and improvement of the gut microbial conditions [14,15,16,17]. Probiotics are defined as “live microorganisms which, when administered in adequate amounts, confer a health benefit on the host” [18], and the modification of the gut microbial composition is one of the main mechanisms underlying the beneficial effects of probiotics on host health [19,20]. Although probiotics have useful effects on human health, useable probiotics are restricted to specific industrial products due to the properties of viable microorganisms.

To date, some research has focused on the beneficial effects of paraprobiotics on human health. Paraprobiotics are defined as nonviable microbial cells (intact or broken) or crude cell extracts, which, when administered (orally or topically) in adequate amounts, confer a benefit on the human or animal consumer [21,22]. Recent studies indicated that paraprobiotics such as *Lactobacillus paracasei* MCC1849 and *Enterococcus faecalis* EC-12 were useful for human health, especially with regard to their immunomodulatory effects [23,24]. Furthermore, paraprobiotics have been shown to have anti-obesity effects in human clinical studies [25,26,27].

*Lactobacillus amylovorus* CP1563 (CP1563) was originally isolated from human fecal specimens. CP1563 was selected as a strain with high ligand activity for peroxisome proliferator-activated receptor α (PPARα) [28], which plays a crucial role in controlling lipid metabolism, reducing adiposity, and improving hepatic steatosis [29]. Previous studies indicated that the effect of CP1563 on obesity prevention was augmented by the fragmentation of the bacterial cells [30]. Furthermore, it was found that the 10-hydroxyoctadecanoic acid (10-HOA) contained in the fragmented CP1563 was the main active compound affecting PPARα ligand activity, and the administration of purified 10-HOA decreased the level of abdominal visceral fat and ameliorated plasma lipid metabolism in diet-induced obese mice [31]. Although the main active component in fragmented CP1563 with regard to obesity prevention was identified as 10-HOA, it remains unclear whether the fragmented CP1563 affects the gut microbial community. While drugs are generally expected to have a therapeutic effect on obesity, food ingredients are expected to have a preventive effect on obesity when taken orally each day. Indeed, many clinical trials evaluating the anti-obesity effects of probiotics or paraprobiotics have found positive results in pre-obese subjects [14,15,25]. In this study, we investigated the effects of fragmented CP1563 on obesity prevention and the gut microbial community in pre-obese subjects.

## 2. Materials and Methods

### 2.1. Study Procedures

The present clinical trial was approved by the Institutional Review Boards of the Medical Corporation Hokubukai Utsukushigaoka Hospital (Sapporo, Japan) and was conducted according to the ethical standards established in the 1964 Declaration of Helsinki. This study was registered with the University Hospital Medical Information Network (UMIN) Clinical Trials Registry as UMIN000033908 (Title: A study to evaluate the effect of test food on body fat-reducing) and was conducted in compliance with the protocol. Written informed consent was obtained from all subjects prior to enrolment. The study was performed from June 2018 to June 2019.

### 2.2. Sample Size

The sample size calculation was performed with G*Power 3.1.9.4 [32], using *t*-tests to determine the difference between two independent groups. Assuming an a priori effect size of 0.42 estimated from our previous study [30], an α error probability of 0.05, and a power (1-β error probability) of 0.80, the resulting total sample size was 180. The analysis exclusion rate was estimated to be 10%; thus, 200 subjects were recruited.

### 2.3. Subjects

The subjects were males and females aged 20 to under 70 years with a body mass index (BMI) from 25.0 to 29.9 kg/m^2^. A BMI from 25.0 to 29.9 kg/m^2^ is defined as “obese (level 1)” by the Japan Society for the Study of Obesity and as “pre-obese” by the World Health Organization [33]. The exclusion criteria were as follows: patients with a history of serious disease; intake of supplements and/or medicines that may affect the results of the study; allergy to test or placebo beverages; pregnancy, lactation, or planning to become pregnant; 60 g or more of alcohol per day on average and/or 20 and more cigarettes a day on average; extremely irregular dietary habits or an alternative work schedule, such as working a midnight shift; and any other reason for ineligibility as judged by the principal investigator. Before participation in the study, blood was collected from all subjects at the hospital (Medical Corporation Shoureikan Sinsapporo Seiryo Hospital, Sapporo, Japan), and all blood analyses were performed at Sapporo Clinical Laboratory Inc. (Sapporo, Japan). Blood analysis was used to confirm the health condition of each subject during the screening phase.

### 2.4. Supplementary Beverages

Test beverages were prepared as previously reported with some modifications [30]. Briefly, *L*. *amylovorus* CP1563 was cultured in a food-grade medium and collected. Bacterial cells were washed, heat-inactivated, and lyophilized. The resulting bacterial powder was fragmented. A previous study indicated that the activity of PPARα in fragmented CP1563 was mainly attributed to 10-hydroxyoctadecanoic acid (10-HOA), and 10-HOA improved lipid metabolism [31]. Therefore, the amount of 10-HOA in the fragmented CP1563 powder was confirmed. Based on a method described in a previous report [31], 1.44 mg of 10-HOA was detected in 200 mg of the fragmented CP1563 powder.

Test beverages with 200 mg of the fragmented CP1563 powder and a placebo beverage without the fragmented CP1563 powder were prepared by blending some food ingredients, pasteurizing the mixture, and packaging the liquid into 500 mL bottles. The nutritional contents of the test and placebo beverages were as follows: The test beverage provided a total of 83.7 kJ (20 kcal/day) kcal, 1.0 g of protein, 0 g of fat, 4.0 g of carbohydrates, and 0.25 g of salt, whereas the placebo beverage provided a total of 83.7 kJ (20 kcal/day) kcal, 1.0 g of protein, 0 g of fat, 4.0 g of carbohydrates, and 0.25 g of salt. The beverages were obtained from Asahi Soft Drinks Co., Ltd. (Tokyo, Japan).

### 2.5. Study Design

This study was designed as a randomized, double-blind, placebo-controlled, parallel group study. The study period consisted of a 2-month screening examination, followed by a 12-week test beverage treatment period. A concealed study coordinator randomly and blindly assigned the 200 subjects into two groups of 100 each that were matched for sex and BMI. Random allocation was conducted by using a computer random number generator. The subjects in one group received the test beverages (a 500 mL bottle of the beverage with the fragmented CP1563 containing 10-HOA per subject per day: CP1563-10-HOA group), and those in the other group received the placebo beverage (a 500 mL bottle of the beverage without the fragmented CP1563 per subject per day: placebo group). All subjects were enrolled in the study prior to random allocation. Allocation to the test or placebo group was concealed from the investigator who enrolled the subjects, the nurses, and the medical doctor in charge of the study.

### 2.6. Measurement

At the start of the treatment period (0 weeks), every 4 weeks (4 and 8 weeks), and at the end of the intervention (12 weeks), we collected the following anthropometric measurements at the hospital (Medical Corporation Shoureikan Sinsapporo Seiryo Hospital, Sapporo, Japan): body weight, BMI, body fat percentage, and waist circumference. Computed tomography (CT) (Bright Speed Elite, GE healthcare, Milwaukee, WI, USA) scans were performed to measure the abdominal visceral fat area (VFA) and subcutaneous fat area (SFA) at baseline, 4, 8, and 12 weeks. To calculate the abdominal VFA, SFA, and total fat area (TFA), which was the sum of the VFA and SFA, the CT scan images were analyzed with Nazca View (AstroStage, Ltd., Tokyo, Japan).

Daily records, which included the consumption of the beverages, dietary content, pedometer measurement, intake of medicines, and physical condition, such as the presence of any symptoms, were recorded by each subject. The intake of total energy, protein, carbohydrate, fat, and salt were calculated by an analysis of the dietary records and using nutrition calculation software (Excel smart nutritional calculation ver.5, Ishiyaku Publishers, Ltd., Tokyo, Japan).

### 2.7. Analysis of Fecal Microbiota

Fecal samples were collected at 0 and 12 weeks, and fecal DNA was extracted by the bead beating method as previously described [34]. Amplification of the 16S rRNA sequence was performed with the primer set of Tru357F (5′-CGCTCTTCCGATCTCTG TACGGRAGGCAGCAG-3′) and Tru806R (5′-CGCTCTTCCGATCTGACGGACTACHVGGGTWTCTAAT-3′) as described previously [35]. The polymerase chain reaction (PCR) products were purified by Agencourt AMPure XP (Beckman Coulter, Inc., Brea, CA, USA), and the products were amplified using the Nextera Index Kit (Illumina, San Diego, CA, USA). After the second PCR, the amplified products were purified using Agencourt AMPure XP. The product was quantified and pooled in an equimolar amount. Sequencing was performed with an Illumina MiSeq system and MiSeq Reagent Kit v.2 (300 Cycle). Sequence data were analyzed as described previously [36]. In brief, Quantitative Insights Into Microbial Ecology (QIIME) ver.1.8.0 was used for sequence filtering and analysis [37]. Quality filtering was performed using fastq files, and sequences with a quality score <29 were removed. Chimeric sequences were removed using USEARCH. Assignment to operational taxonomic units (OTUs) was performed using open-reference OTU picking with a 97% threshold for pairwise identity. After removing the OTUs containing < 5 sequences, the OTUs were classified taxonomically using the Greengenes reference database [38].

### 2.8. Measurement of Fecal Short Chain Fatty Acids (SCFAs)

Fecal samples were collected at 0 and 12 weeks. Using high-performance liquid chromatography with a pH indicator (LaChrom Elite; Hitachi High-Technologies Corporation, Tokyo, Japan), the concentrations of acetic acid, propionic acid, n-butyric acid, iso-butyric acid, n-valeric acid, and iso-valeric acid were measured as reported in a previous report [39].

### 2.9. Statistical Analysis

Unpaired Student’s *t*-tests, Dunnett’s test, and the χ^2^ test were performed using JMP13 (SAS Institute Japan Ltd., Tokyo, Japan). The data are presented as the mean ± standard error of the mean (SEM). For the analysis of differences between groups, principal coordinate analysis (PCoA) and permutational multivariate analysis of variance (MANOVA) using distance matrices based on the Bray–Curtis distance were performed with R software (ver. 4.0.0) with the vegan package. PCoA and permutational MANOVA based on weighted and unweighted UniFrac distance, and α-diversity analyses such as chao1, Shannon, and PD whole tree were run on QIIME software (ver. 1.8.0). In the comparison of gut microbial compositional change values, *p* values were adjusted for multiple testing using the false discovery rate (FDR) with the qvalue package of R software (ver. 4.0.0) and a parameter (lambda = 0.5) [40]. Differences were considered significant at *p* < 0.05 or *q* < 0.05.

## 3. Results

### 3.1. Characteristics of the Subjects

**Figure 1:**
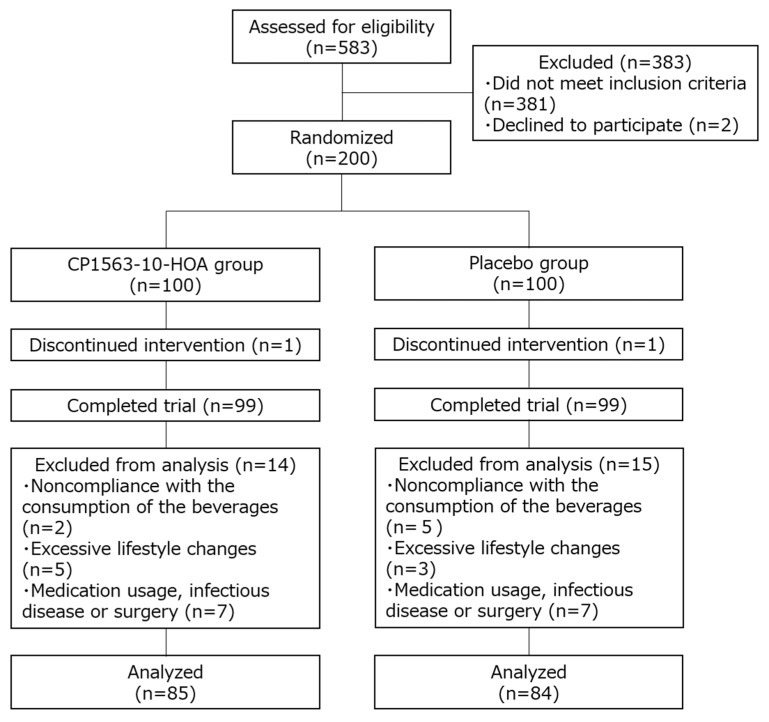
Flow diagram of the progress stages of this study.

The process for the selection of the subjects enrolled in this study is shown in Figure 1. In accordance with the inclusion and exclusion criteria, 385 of the 585 subjects were excluded, and 200 subjects (110 women and 90 men) were deemed eligible. The subjects were considered healthy by a principal investigator based on the results of the blood analysis, blood pressure measurement, and self-report. The subjects were enrolled and randomly assigned into the CP1563-10-HOA group or the placebo group (100 subjects per group) by the study coordinator. Two subjects dropped out of the study because of personal problems not related to the study. After the intervention, a total of 29 subjects were excluded by the principal investigator because of their inability to comply with the study rules: 7 subjects were noncompliant with the consumption of the beverages; 8 subjects had excessive lifestyle changes; and 14 subjects took medicine (antibiotics or medical drugs) or had an infectious disease or surgery that could have affected the results. Finally, the analysis was conducted with 169 subjects (85 subjects in the CP1563-10-HOA group and 84 subjects in the placebo group). There were no significant differences in age, male/female ratio, BMI, abdominal fat area, body fat percentage, or waist circumference between the CP1563-10-HOA and placebo groups (Table 1).

### 3.2. Dietary Nutritional Content and Physical Activity During the Study Period

Salt intake was significantly higher in the CP1563-10-HOA group than in the placebo group at 0 weeks; however, the intake of other nutrients (calories, protein, fat, carbohydrate) as calculated by nutrition calculation software and daily physical activity as defined by the pedometer reading were not significantly different between the two groups during the study period (Table 2). The test beverage consumption rates were 97.1 ± 0.5% and 98.0 ± 0.4% in the CP1563-10-HOA and the placebo groups, respectively. No adverse events were observed by the principal investigators in the daily self-reports and interviews during the study period.

### 3.3. Effects of Fragmented CP1563 on Abdominal Fat Area, Body Weight, and BMI

**Figure 2:**
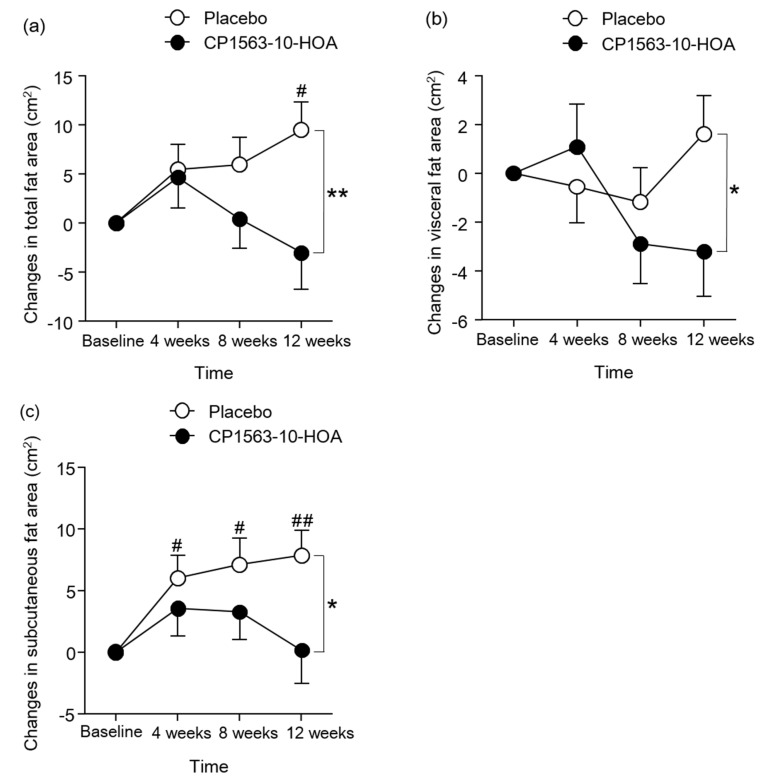
Changes in abdominal fat areas. Changes in total fat area (**a**), changes in visceral fat area (**b**), and changes in subcutaneous fat area (**c**). Data are shown as the mean ± SEM (the CP1563-10-HOA group: n = 85, the placebo group: n = 84). * *p* < 0.05, ** *p* < 0.01. vs. placebo group (by Student’s *t*-test). ^#^ *p* < 0.05, ^##^ *p* < 0.01. vs. baseline (by Dunnett’s test).

**Figure 3:**
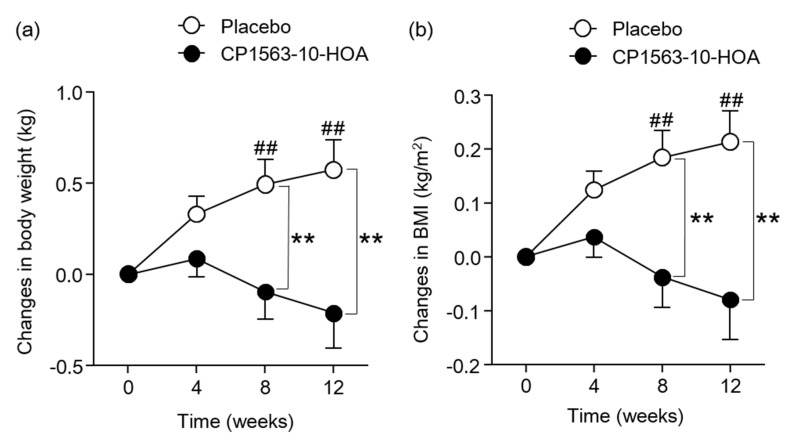
Changes in anthropometric parameters. Changes in body weight (**a**) and changes in BMI (**b**). Data are shown as the mean ± SEM (the CP1563-10-HOA group: n = 85, the placebo group: n = 84). ** *p* < 0.01. vs. placebo group (by Student’s *t*-test.). ^##^ *p* < 0.01. vs. 0 weeks (by Dunnett’s test).

Figure 2 shows the changes in abdominal fat area. The changes in TFA, VFA, and SFA at 12 weeks were significantly smaller in the CP1563-10-HOA group than in the control group. The changes in TFA of the placebo group was increased significantly at 12 weeks compared to the baseline, and the changes in SFA of the placebo group were increased significantly at 4, 8, and 12 weeks compared to the baseline. The body weight in the CP1563-10-HOA group at 12 weeks was significantly lower than that in the placebo group (Table S1). Figure 3 shows the changes in anthropometric parameters. The changes in body weight and BMI at 8 and 12 weeks were significantly smaller in the CP1563-10-HOA group than in the placebo group. The changes in body weight and BMI of the placebo group were increased significantly at 8 and 12 weeks compared to 0 weeks. The body fat percentage and waist circumference were not significantly different between the two groups during the study period (Table S1).

### 3.4. Effect of Fragmented CP1563 on Fecal Microbiota and Fecal SCFAs

**Figure 4:**
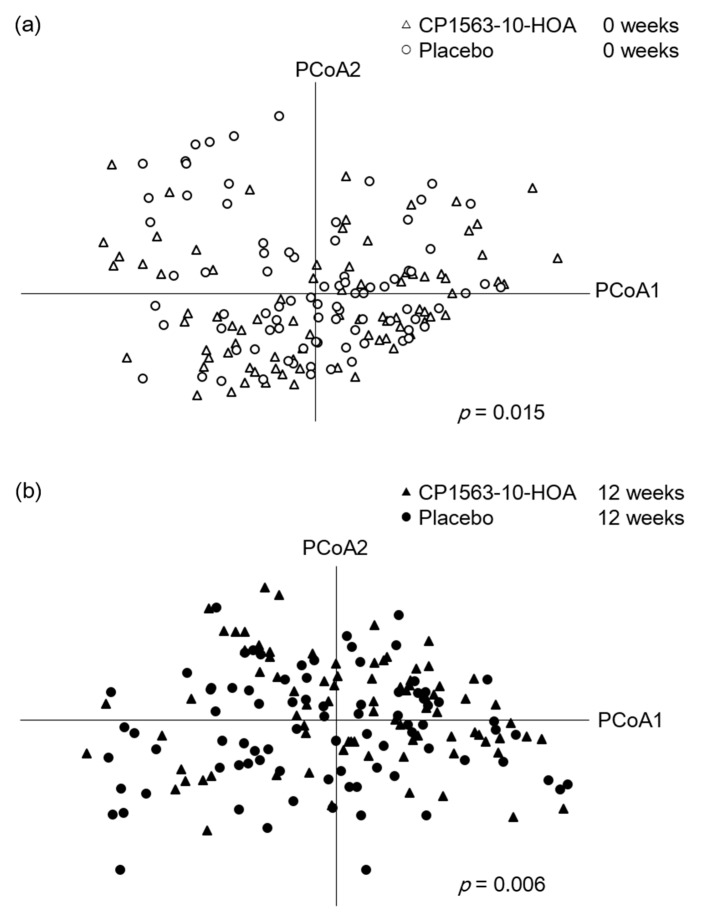
Plots of the placebo and CP1563-10-HOA groups at 0 (**a**) and 12 (**b**) weeks were visualized by principal coordinate analysis (PCoA) based on the Bray–Curtis distance using the bacterial taxa at the genus level (the CP1563-10-HOA group at 0 weeks: n = 83, at 12 weeks: n = 82; the placebo group at 0 weeks: n = 84, at 12 weeks: n = 83). The *p* values were calculated by permutational MANOVA based on the Bray–Curtis distance using the bacterial taxa at the genus level.

Because 6 samples were not submitted by subjects, 83 samples from the CP1563-10-HOA group at 0 weeks, 82 samples from the CP1563-10-HOA group at 12 weeks, 84 samples from the placebo group at 0 weeks, and 83 samples from the placebo group at 12 weeks were analyzed. The α-diversity analyses (chao1, Shannon, and PD whole tree) showed no significant difference between the two groups at each timepoint (Table S2). The fecal microbiota profiles of the CP1563-10-HOA group and the placebo group based on the Bray–Curtis distance using bacterial taxa at the genus level, weighted UniFrac distance, and unweighted UniFrac distance were visualized by PCoA analysis and analyzed by permutational MANOVA (Figure 4, Figures S1 and S2). The result of the permutational MANOVA revealed significant differences between the two groups at 0 (*p* = 0.015) and 12 (*p* = 0.006) weeks. The analysis based on the weighted UniFrac distance were in accord with the analysis based on the Bray–Curtis distance. The analysis based on the unweighted UniFrac distance showed a significant difference between two groups at 12 weeks; however, no significant difference was observed at 0 weeks.

Because a significant difference in the microbial community between the two groups was observed at 0 weeks, we analyzed the change values calculated by subtracting the taxon abundance at week 0 from the taxon abundance at week 12 to analyze the effect of the test beverages on the gut microbiota. The taxon abundances that averaged over 1% in a total of 332 samples were used in the statistical analysis to evaluate the effect on the predominant taxa in the fecal microbiota (Table S3). The change values of fecal microbial abundances at the genus level are summarized in Table 3 (the CP1563-10-HOA group: n = 80, the placebo group: n = 83). The analysis revealed that the changes in *Roseburia* and *Lachnospiraceae;g* were significantly greater in the CP1563-10-HOA group than in the placebo group, and the changes in *Collinsella* were significantly smaller in the CP1563-10-HOA group than in the placebo group. 

Because 1 sample was not submitted by a subject, 85 samples from the CP1563-10-HOA group at 0 weeks, 84 samples from the CP1563-10-HOA group at 12 weeks, 84 samples from the placebo group at 0 weeks, and 84 samples from the placebo group at 12 weeks were analyzed to determine fecal SCFA concentrations (Table 4). The changes in the concentrations of fecal SCFAs (acetic acid, propionic acid, n-butyric acid, iso-butyric acid, n-valeric acid, and iso-valeric acid) were not significantly different between the two groups.

## 4. Discussion

Although gut microbial conditions need to be taken into account when attempting to prevent obesity, it remains unclear whether fragmented CP1563 affects the gut microbial community. In this study, we investigated the effects of fragmented CP1563 on obesity prevention and the gut microbial community in pre-obese subjects.

The TFA, VFA, and SFA at 12 weeks were significantly lower in the CP1563-10-HOA group than in the control group. Body weight and BMI were also significantly lower in the CP1563-10-HOA group than in the placebo group. It is known that abdominal fat reduction is mainly influenced by a reduction in nutrient intake and an increase in physical activity. In this study, nutrient intake (calories, protein, fat, carbohydrate) as calculated by nutrition calculation software and daily physical activity as defined by pedometer readings were not significantly different between the two groups. These results suggested that fragmented CP1563 reduced abdominal fat area, body weight, and BMI in pre-obese subjects. Several human clinical trials showed an anti-obesity effect of the intake of paraprobiotics [25,26,27]. The anti-obesity effect of fragmented CP1563 in this study supported the results of a previous study, which showed the anti-obesity effect of fragmented CP1563 [30]. The obesity conditions in the control group alone worsened during the intervention (Figure 2 and Figure 3). Previous studies also indicated that obesity conditions in pre-obese adults worsened during the study period [15,25]. Obesity might continue to worsen in pre-obese adults unless they consciously try to improve their current lifestyles. However, whether these findings apply to all pre-obese Japanese individuals remains unclear. Even in such a situation, CP1563 had a beneficial effect on obesity prevention in the pre-obese subjects.

Few findings have focused on the effects of the oral intake of paraprobiotics on the gut microbial community in obesity prevention [26,27]. In addition, there is no evidence about the effects of fragmented CP1563 on the gut microbial community. The fecal microbiota profiles analyzed by permutational MANOVA using the unweighted UniFrac distance showed a significant difference between the two groups only at 12 weeks (Figure S2). However, the fecal microbiota profiles analyzed by permutational MANOVA using the Bray–Curtis and weighted UniFrac distance showed a significant difference between the two groups at 0 and 12 weeks (Figure 4, Figure S1). It is known that the unweighted UniFrac distance is a qualitative distance matrix and that the weighted UniFrac and Bray–Curtis distances are quantitative distance matrices. These results suggested that ingestion of the fragmented CP1563 has the potential to have a qualitative effect on the gut microbial community, such as the frequency of the bacterial taxa. However, the initial difference in the gut microbial community between the two groups made it difficult to evaluate the effects of ingesting the fragmented CP1563 on the quantitative effects, such as the abundance of the predominant bacterial taxa. For evaluation of the effect on the predominant taxa in the fecal microbiota by the ingestion of the fragmented CP1563, compositional changes in the fecal microbiota were analyzed. The analysis revealed that the changes in *Lachnospiraceae;g* and *Roseburia* were significantly greater and the changes in *Collinsella* was significantly smaller in the CP1563-10-HOA group than in the placebo group. *Lachnospiraceae;g* is an unknown genus belonging to the family *Lachnospiraceae*. Because some butyrate-producing bacteria, such as *Eubacterium rectale* and *Eubacterium hallii,* belong to *Lachnospiraceae* [41], some species belonging to *Lachnospiraceae;g* have the potential to be butyrate-producing bacteria. Some bacterial species of the genera *Roseburia* such as *Roseburia intestinalis* are known to be butyric acid-producing bacteria [42]. It is possible that an increase in the abundance of butyrate-producing bacteria in the digestive tract leads to an increase in butyrate levels. To understand the Japanese gut microbiota, these microbiota from healthy subjects are summarized in Table S4. Because the composition of the gut microbiota was different in each study, discussing the characteristics of the gut microbiota in this study was thought to be difficult. However, the abundance of genus *Bacteroides*, which is known to be reduced in obesity-related conditions [43], might be small in this study.

Butyrate is one of the main SCFAs in the human digestive tract, along with propionate and acetate. Butyrate is an activator of GPR41, which acts as an SCFA receptor [44,45]. GPR41 activation increases heart rate and energy expenditure through sympathetic activation [46], and GPR41 activation induces the secretion of PYY- and GLP-1-through endocrine L-cells [47]. Butyrate also activates GPR43, which is highly expressed in adipose tissue [48]. The activation of GPR43 inhibits fat accumulation in adipose tissue by suppressing insulin signaling in adipocytes [48]. Another study revealed that an increase in microbial-derived butyrate induced the differentiation of colonic regulatory T cells through epigenetic modification, and the induction of regulatory T cells showed an anti-inflammatory effect [49]. Several studies have shown that the adipose tissue of obese organisms exhibits chronic low-grade inflammation [50,51], and upregulating anti-inflammatory immune activities by increasing the level of butyrate has the potential to affect obesity. Based on these insights, the increase in the level of butyrate is thought to be involved in reducing obesity.

This study evaluated the changes in fecal SCFA concentrations. However, no significant differences were observed in the changes in fecal SCFA concentrations between the two groups. SCFAs are utilized as an energy source for the colorectal epithelium [52,53] and are rapidly absorbed from the intestine and subsequently utilized by the host as a substrate for metabolic energy production [54]. In addition, a previous review proposed that it is necessary to measure portal appearance as well as portal drained viscera metabolism to assess the quantitative and qualitative contribution of SCFA and SCFA metabolites to whole-animal metabolism [55]. Evaluations of the SCFA levels in the serum and organs to determine the effect of SCFAs on host metabolism are needed; however, there were no data collected on serum and organ SCFA levels in our study. Therefore, further studies are needed to determine the relationship between the increases in butyrate-producing bacteria and butyrate levels.

The abundance of the genus *Roseburia* was negatively correlated with the plasma glucose levels in a human study [56], and the depletion of the genus *Roseburia* is a characteristic feature of the microbiome in type 2 diabetic patients [57]. In addition, a high abundance of the genus *Collinsella* is a characteristic feature of the microbiome in type 2 diabetic patients [58], and a structured weight loss program reduced the abundance of the genus *Collinsella* in obese type 2 diabetic patients [59]. Based on these insights, the alterations in the abundances of *Roseburia* and *Collinsella* observed in our study could have affected obesity in these patients. It is known that butyrate-producing bacteria are reduced in abundance under inflammatory conditions such as in inflammatory bowel disease [60]. Although there is a possibility that a reduction in obesity may lead to the alleviation of inflammatory conditions in the host and an increase in the abundance of butyrate-producing bacteria in the gut microbial environment, it remains unclear whether the alterations of fecal microbiota abundances were affected by the oral intake of the fragmented CP1563 cells or by the host response to the improvement in the pre-obese condition. Because a significant difference in the gut microbial community between the two groups was observed at 0 weeks, future clinical trials with no difference in the gut microbial community at 0 weeks are needed for an accurate determination of the relationship between the oral intake of fragmented CP1563 and the gut microbial community.

In summary, the daily ingestion of the fragmented CP1563 containing 10-HOA had a beneficial effect on obesity prevention in pre-obese healthy subjects. Our results suggested that fragmented CP1563 containing 10-HOA affected the gut microbial community. Our findings provide new insights into the anti-obesity effects of this paraprobiotic strain.

## Figures and Tables

**Table 1:** Characteristics of subjects.

Parameters	CP1563-10-HOA Group	Placebo Group	*p* Value
Age (years)	43.6 ± 1.3	41.7 ± 1.5	0.34
Sex (male/female)	38/47	41/43	0.59
Height (cm)	163.5 ± 0.9	165.7 ± 1.0	0.10
Body weight (kg)	72.5 ± 0.9	74.5 ± 1.0	0.14
Body mass index (BMI) (kg/m^2^)	27.0 ± 0.2	27.1 ± 0.2	0.86
Abdominal total fat area (cm^2^)	335.0 ± 6.7	340.1± 6.3	0.58
Abdominal visceral fat area (cm^2^)	92.0 ± 3.5	90.8 ± 3.7	0.81
Abdominal subcutaneous fat area (cm^2^)	243.0 ± 6.5	249.3 ± 6.4	0.48
Body fat percentage (%)	34.2 ± 0.7	33.9 ± 0.7	0.79
Waist circumference (cm)	91.1 ± 0.7	91.4 ± 0.7	0.74

Values are mean ± SEM (the CP1563-10-HOA group: *n* = 85, the placebo group: *n* = 84). All *p* values except for the male/female ratio were calculated by Student’s t-test. The χ^2^ test was used for analysis of the male/female ratio.

**Table 2:** Daily nutrient intake and physical activity.

Parameters	Treatment	Values			
		0 Weeks	4 Weeks	8 Weeks	12 Weeks
Calories (kcal/day)	CP1563-10-HOA	1748.8 ± 49.6	1691.7 ± 53.6	1669.8 ± 56.4	1624.9 ± 63.7
	Placebo	1652.9 ± 54.1	1644.5 ± 57.2	1583.3 ± 56.4	1651.6 ± 60.9
	*p* value (vs. placebo)	0.19	0.55	0.28	0.76
Protein (g/day)	CP1563-10-HOA	66.5 ± 2.2	66.7 ± 2.7	62.8 ± 2.1	63.5 ± 3.0
	Placebo	62.5 ± 2.3	63.1 ± 2.4	58.7 ± 2.6	68.0 ± 7.0
	*p* value (vs. placebo)	0.21	0.32	0.21	0.56
Fat (g/day)	CP1563-10-HOA	62.4 ± 2.1	57.9 ± 2.3	59.4 ± 2.3	56.4 ± 2.5
	Placebo	59.2 ± 2.6	57.8 ± 2.5	56.9 ± 2.4	60.7 ± 2.7
	*p* value (vs. placebo)	0.34	0.98	0.44	0.25
Carbohydrate (g/day)	CP1563-10-HOA	218.9 ± 7.2	219.3 ± 8.6	210.9 ± 8.5	206.4 ± 8.4
	Placebo	212.0 ± 7.9	218.1 ± 8.1	204.0 ± 8.2	207.5 ± 8.2
	*p* value (vs. placebo)	0.52	0.92	0.56	0.93
Salt (g/day)	CP1563-10-HOA	9.1 ± 0.3	8.4 ± 0.4	9.0 ± 0.4	8.2 ± 0.4
	Placebo	8.1 ± 0.3	8.2 ± 0.3	9.1 ± 0.4	8.3 ± 0.3
	*p* value (vs. placebo)	0.03	0.66	0.77	0.88
Steps (counts/day)	CP1563-10-HOA	6025.9 ± 315.0	5551.9 ± 299.8	5833.0 ± 338.2	6414.5 ± 498.2
	Placebo	5979.6 ± 330.4	6691.7 ± 839.6	6957.9 ± 906.6	7270.5 ± 866.1
	*p* value (vs. placebo)	0.92	0.20	0.24	0.39

Values are the mean ± SEM (the CP1563-10-HOA group: *n* = 85, the placebo group: *n* = 84). The *p* values were calculated by Student’s *t*-test.

**Table 3:** Compositional change values of the fecal microbiota at the genus level.

Genus	Compositional Change (%)		*p* Value	*q* Value
	CP1563-10-HOA	Placebo		
*Blautia*	−2.1 ± 1.2	−0.8 ± 1.0	0.39	0.45
*Bifidobacterium*	−0.0 ± 1.2	1.3 ± 1.3	0.44	0.45
*Faecalibacterium*	0.8 ± 1.1	2.0 ± 1.1	0.45	0.45
*Coprococcus*	−1.1 ± 0.6	−1.3 ± 0.7	0.80	0.57
*Lachnospiraceae;*g*_[Ruminococcus]*	−0.7 ± 0.5	−1.3 ± 0.6	0.43	0.45
*Lachnospiraceae;*g	1.0 ± 0.4	−0.6 ± 0.5	0.01	0.047
*Roseburia*	0.8 ± 0.7	−1.4 ± 0.6	0.01	0.047
*Bacteroides*	1.5 ± 1.3	2.3 ± 1.0	0.64	0.53
*Streptococcus*	−0.8 ± 0.5	0.2 ± 0.6	0.21	0.37
*Ruminococcus*	0.4 ± 0.4	−0.3 ± 0.3	0.22	0.37
*Dorea*	−0.1 ± 0.2	−0.2 ± 0.2	0.88	0.59
*Erysipelotrichaceae;*g*_[Eubacterium]*	−0.0 ± 0.2	−0.1 ± 0.2	0.80	0.57
*Lachnospiraceae;Other*	0.1 ± 0.1	0.0 ± 0.1	0.62	0.53
*Butyricicoccus*	0.0 ± 1.1	−0.4 ± 0.1	0.046	0.12
*Collinsella*	−0.3 ± 0.1	0.2 ± 0.1	0.003	0.03

Values are the mean ± SEM (the CP1563-10-HOA group: *n* = 80, the placebo group: *n* = 83). The *p* values were calculated by Student’s *t*-test and adjusted using the false discovery rate (FDR) method. FDR-adjusted *p* values (*q* values) are listed.

**Table 4:** Changes in the concentrations of fecal short chain fatty acids (SCFAs).

SCFAs	Change in the Concentration (mg g^−1^ Feces)		*p* Value
	CP1563-10-HOA	Placebo	
Acetic acid	7.9 ± 2.1	4.7 ± 1.7	0.23
Propionic acid	2.4 ± 0.8	1.0 ± 0.8	0.22
n-Butyric acid	0.8 ± 0.8	1.1 ± 0.7	0.78
iso-Butyric acid	0.5 ± 0.1	0.2 ± 0.1	0.10
n-Valeric acid	0.2 ± 0.1	0.3 ± 0.2	0.72
iso-Valeric acid	0.2 ± 0.2	0.4 ± 0.2	0.39

Values are the mean ± SEM (the CP1563-10-HOA group: n = 84, the placebo group: n = 84). The *p* values were calculated by Student’s *t*-test.

## Supplementary Materials

The following are available online at https://www.mdpi.com/2076-2607/8/2/304/s1, Figure S1: The fecal microbiota profiles of the CP1563-10-HOA group and placebo group based on weighted UniFrac distance visualized by PCoA analysis, Figure S2: The fecal microbiota profiles of the CP1563-10-HOA group and placebo group based on unweighted UniFrac distance visualized by PCoA analysis, Table S1: Abdominal fat areas and anthropometric parameters, Table S2: The α-diversity indices, Table S3: Components of the fecal microbiota at the genus level, Table S4: Summary of the characteristics of subjects and gut microbial composition of this study at week 0 and other studies.

## References

[1] Mathieu P., Pibarot P., Despres J.P. (2006). Metabolic syndrome: The danger signal in atherosclerosis. Vasc. Health Risk Manag..

[2] Sharma A.M., Chetty V.T. (2005). Obesity, hypertension and insulin resistance. Acta Diabetol..

[3] Simopoulos A.P. (2013). Dietary omega-3 fatty acid deficiency and high fructose intake in the development of metabolic syndrome, brain metabolic abnormalities, and non-alcoholic fatty liver disease. Nutrients.

[4] Wu G.D., Chen J., Hoffmann C., Bittinger K., Chen Y.-Y., Keilbaugh S.A., Bewtra M., Knights D., Walters W.A., Knight R. (2011). Linking long-term dietary patterns with gut microbial enterotypes. Sciences.

[5] Shen T.D. (2017). Diet and Gut Microbiota in Health and Disease. Nestle Nutr. Inst. Workshop Ser..

[6] Turnbaugh P.J., Backhed F., Fulton L., Gordon J.I. (2008). Diet-induced obesity is linked to marked but reversible alterations in the mouse distal gut microbiome. Cell Host Microbe.

[7] Turnbaugh P.J., Ridaura V.K., Faith J.J., Rey F.E., Knight R., Gordon J.I. (2009). The effect of diet on the human gut microbiome: A metagenomic analysis in humanized gnotobiotic mice. Sci. Transl. Med..

[8] Cani P.D., Bibiloni R., Knauf C., Waget A., Neyrinck A.M., Delzenne N.M., Burcelin R. (2008). Changes in gut microbiota control metabolic endotoxemia-induced inflammation in high-fat diet-induced obesity and diabetes in mice. Diabetes.

[9] De La Serre C.B., Ellis C.L., Lee J., Hartman A.L., Rutledge J.C., Raybould H.E. (2010). Propensity to high-fat diet-induced obesity in rats is associated with changes in the gut microbiota and gut inflammation. Am. J. Physiol. Gastrointest. Liver Physiol..

[10] Ding S., Chi M.M., Scull B.P., Rigby R., Schwerbrock N.M., Magness S., Jobin C., Lund P.K. (2010). High-fat diet: Bacteria interactions promote intestinal inflammation which precedes and correlates with obesity and insulin resistance in mouse. PLoS ONE.

[11] Morimoto-Kobayashi Y., Ohara K., Ashigai H., Kanaya T., Koizumi K., Manabe F., Kaneko Y., Taniguchi Y., Katayama M., Kowatari Y. (2016). Matured hop extract reduces body fat in healthy overweight humans: A randomized, double-blind, placebo-controlled parallel group study. Nutr. J..

[12] Racine N.M., Watras A.C., Carrel A.L., Allen D.B., McVean J.J., Clark R.R., O’Brien A.R., O’Shea M., Scott C.E., Schoeller D.A. (2010). Effect of conjugated linoleic acid on body fat accretion in overweight or obese children. Am. J. Clin. Nutr..

[13] Snitker S., Fujishima Y., Shen H., Ott S., Pi-Sunyer X., Furuhata Y., Sato H., Takahashi M. (2009). Effects of novel capsinoid treatment on fatness and energy metabolism in humans: Possible pharmacogenetic implications. Am. J. Clin. Nutr..

[14] Kadooka Y., Sato M., Imaizumi K., Ogawa A., Ikuyama K., Akai Y., Okano M., Kagoshima M., Tsuchida T. (2010). Regulation of abdominal adiposity by probiotics (Lactobacillus gasseri SBT2055) in adults with obese tendencies in a randomized controlled trial. Eur. J. Clin. Nutr..

[15] Minami J., Iwabuchi N., Tanaka M., Yamauchi K., Xiao J.Z., Abe F., Sakane N. (2018). Effects of Bifidobacterium breve B-3 on body fat reductions in pre-obese adults: A randomized, double-blind, placebo-controlled trial. Biosci. Microbiota Food Health.

[16] O’Toole P.W., Cooney J.C. (2008). Probiotic bacteria influence the composition and function of the intestinal microbiota. Interdiscip. Perspect. Infect. Dis..

[17] Takahashi S., Anzawa D., Takami K., Ishizuka A., Mawatari T., Kamikado K., Sugimura H., Nishijima T. (2016). Effect of Bifidobacterium *Animalis* ssp. lactis GCL2505 on visceral fat accumulation in healthy Japanese adults: A randomized controlled trial. Biosci. Microbiota Food Health.

[18] Food and Agriculture Organization of the United Nations/World Health Organization (FAO/WHO) (2002). Guidelines for the Evaluation of Probiotics in Food. Joint FAO/WHO Working Group on Drafting Guidelines for the Evaluation of Probiotics in Food.

[19] Bagarolli R.A., Tobar N., Oliveira A.G., Araujo T.G., Carvalho B.M., Rocha G.Z., Vecina J.F., Calisto K., Guadagnini D., Prada P.O. (2017). Probiotics modulate gut microbiota and improve insulin sensitivity in DIO mice. J. Nutr. Biochem..

[20] Murphy E.F., Cotter P.D., Hogan A., O’Sullivan O., Joyce A., Fouhy F., Clarke S.F., Marques T.M., O’Toole P.W., Stanton C. (2013). Divergent metabolic outcomes arising from targeted manipulation of the gut microbiota in diet-induced obesity. Gut.

[21] Deshpande G., Athalye-Jape G., Patole S. (2018). Para-probiotics for Preterm Neonates—The Next Frontier. Nutrients.

[22] Taverniti V., Guglielmetti S. (2011). The immunomodulatory properties of probiotic microorganisms beyond their viability (ghost probiotics: Proposal of paraprobiotic concept). Genes Nutr..

[23] Murata M., Kondo J., Iwabuchi N., Takahashi S., Yamauchi K., Abe F., Miura K. (2018). Effects of paraprobiotic Lactobacillus paracasei MCC1849 supplementation on symptoms of the common cold and mood states in healthy adults. Benef. Microbes.

[24] Nishibayashi R., Inoue R., Harada Y., Watanabe T., Makioka Y., Ushida K. (2015). RNA of Enterococcus faecalis Strain EC-12 Is a Major Component Inducing Interleukin-12 Production from Human Monocytic Cells. PLoS ONE.

[25] Higashikawa F., Noda M., Awaya T., Danshiitsoodol N., Matoba Y., Kumagai T., Sugiyama M. (2016). Antiobesity effect of Pediococcus pentosaceus LP28 on overweight subjects: A randomized, double-blind, placebo-controlled clinical trial. Eur. J. Clin. Nutr..

[26] Hsieh M.C., Tsai W.H., Jheng Y.P., Su S.L., Wang S.Y., Lin C.C., Chen Y.H., Chang W.W. (2018). The beneficial effects of Lactobacillus reuteri ADR-1 or ADR-3 consumption on type 2 diabetes mellitus: A randomized, double-blinded, placebo-controlled trial. Sci. Rep..

[27] Pedret A., Valls R.M., Calderon-Perez L., Llaurado E., Companys J., Pla-Paga L., Moragas A., Martin-Lujan F., Ortega Y., Giralt M. (2019). Effects of daily consumption of the probiotic Bifidobacterium animalis subsp. lactis CECT 8145 on anthropometric adiposity biomarkers in abdominally obese subjects: A randomized controlled trial. Int. J. Obes..

[28] Nakamura F., Ishida Y., Sawada D., Ashida N., Sugawara T., Sakai M., Goto T., Kawada T., Fujiwara S. (2016). Fragmented Lactic Acid Bacterial Cells Activate Peroxisome Proliferator-Activated Receptors and Ameliorate Dyslipidemia in Obese Mice. J. Agric. Food Chem..

[29] Evans R.M., Barish G.D., Wang Y.X. (2004). PPARs and the complex journey to obesity. Nat. Med..

[30] Nakamura F., Ishida Y., Aihara K., Sawada D., Ashida N., Sugawara T., Aoki Y., Takehara I., Takano K., Fujiwara S. (2016). Effect of fragmented Lactobacillus amylovorus CP1563 on lipid metabolism in overweight and mildly obese individuals: A randomized controlled trial. Microb. Ecol. Health Dis..

[31] Aoki Y., Sugawara T., Yanagihara S., Goto T., Kawada T., Fujiwara S., Sawada D. (2018). 10-Hydroxyoctadecanoic Acid from Lactobacillus amylovorus CP1563 Activates PPARα and Improves Dyslipidemia. Pharmacometrics.

[32] Faul F., Erdfelder E., Lang A.-G., Buchner A. (2007). G*Power 3: A flexible statistical power analysis program for the social, behavioral, and biomedical sciences. Behav. Res. Methods.

[33] World Health Organization (2000). Obesity: Preventing and Managing the Global Epidemic. Report of a WHO Consultation.

[34] Hatanaka M., Yamamoto K., Suzuki N., Iio S., Takara T., Morita H., Takimoto T., Nakamura T. (2018). Effect of Bacillus subtilis C-3102 on loose stools in healthy volunteers. Benef. Microbes.

[35] Imoto N., Morita H., Amanuma F., Maruyama H., Watanabe S., Hashiguchi N. (2018). Maternal antimicrobial use at delivery has a stronger impact than mode of delivery on bifidobacterial colonization in infants: A pilot study. J. Perinatol..

[36] Sawada D., Kuwano Y., Tanaka H., Hara S., Uchiyama Y., Sugawara T., Fujiwara S., Rokutan K., Nishida K. (2019). Daily intake of Lactobacillus gasseri CP2305 relieves fatigue and stress-related symptoms in male university Ekiden runners: A double-blind, randomized, and placebo-controlled clinical trial. J. Funct. Foods.

[37] Caporaso J.G., Kuczynski J., Stombaugh J., Bittinger K., Bushman F.D., Costello E.K., Fierer N., Pena A.G., Goodrich J.K., Gordon J.I. (2010). QIIME allows analysis of high-throughput community sequencing data. Nat. Methods.

[38] The Greengenes Reference Database. https://greengenes.secondgenome.com/.

[39] Ikeda N., Saito Y., Shimizu J., Ochi A., Mizutani J., Watabe J. (1994). Variations in concentrations of bacterial metabolites, enzyme activities, moisture, pH and bacterial composition between and within individuals in faeces of seven healthy adults. J. Appl. Bacteriol..

[40] Storey J.D. (2002). A direct approach to false discovery rates. J. R. Stat. Soc. Ser. B.

[41] Duncan S.H., Louis P., Flint H.J. (2004). Lactate-utilizing bacteria, isolated from human feces, that produce butyrate as a major fermentation product. Appl. Environ. Microbiol..

[42] Pryde S.E., Duncan S.H., Hold G.L., Stewart C.S., Flint H.J. (2002). The microbiology of butyrate formation in the human colon. FEMS Microbiol. Lett..

[43] Ley R.E., Turnbaugh P.J., Klein S., Gordon J.I. (2006). Microbial ecology: Human gut microbes associated with obesity. Nature.

[44] Brown A.J., Goldsworthy S.M., Barnes A.A., Eilert M.M., Tcheang L., Daniels D., Muir A.I., Wigglesworth M.J., Kinghorn I., Fraser N.J. (2003). The Orphan G protein-coupled receptors GPR41 and GPR43 are activated by propionate and other short chain carboxylic acids. J. Biol. Chem..

[45] Le Poul E., Loison C., Struyf S., Springael J.Y., Lannoy V., Decobecq M.E., Brezillon S., Dupriez V., Vassart G., Van Damme J. (2003). Functional characterization of human receptors for short chain fatty acids and their role in polymorphonuclear cell activation. J. Biol. Chem..

[46] Inoue D., Kimura I., Wakabayashi M., Tsumoto H., Ozawa K., Hara T., Takei Y., Hirasawa A., Ishihama Y., Tsujimoto G. (2012). Short-chain fatty acid receptor GPR41-mediated activation of sympathetic neurons involves synapsin 2b phosphorylation. FEBS Lett..

[47] Tazoe H., Otomo Y., Karaki S., Kato I., Fukami Y., Terasaki M., Kuwahara A. (2009). Expression of short-chain fatty acid receptor GPR41 in the human colon. Biomed. Res..

[48] Kimura I., Inoue D., Hirano K., Tsujimoto G. (2014). The SCFA Receptor GPR43 and Energy Metabolism. Front. Endocrinol..

[49] Furusawa Y., Obata Y., Fukuda S., Endo T.A., Nakato G., Takahashi D., Nakanishi Y., Uetake C., Kato K., Kato T. (2013). Commensal microbe-derived butyrate induces the differentiation of colonic regulatory T cells. Nature.

[50] Lumeng C.N., Saltiel A.R. (2011). Inflammatory links between obesity and metabolic disease. J. Clin. Investig..

[51] Saltiel A.R., Olefsky J.M. (2017). Inflammatory mechanisms linking obesity and metabolic disease. J. Clin. Investig..

[52] Bergman E.N. (1990). Energy contributions of volatile fatty acids from the gastrointestinal tract in various species. Physiol. Rev..

[53] Roediger W.E. (1980). Role of anaerobic bacteria in the metabolic welfare of the colonic mucosa in man. Gut.

[54] Nakatani M., Inoue R., Tomonaga S., Fukuta K., Tsukahara T. (2018). Production, Absorption, and Blood Flow Dynamics of Short-Chain Fatty Acids Produced by Fermentation in Piglet Hindgut during the Suckling(-)Weaning Period. Nutrients.

[55] Kristensen N.B., Danfaer A., Agergaard N. (1998). Absorption and metabolism of short-chain fatty acids in ruminants. Arch. Tierernahr..

[56] Larsen N., Vogensen F.K., van den Berg F.W., Nielsen D.S., Andreasen A.S., Pedersen B.K., Al-Soud W.A., Sorensen S.J., Hansen L.H., Jakobsen M. (2010). Gut microbiota in human adults with type 2 diabetes differs from non-diabetic adults. PLoS ONE.

[57] Qin J., Li Y., Cai Z., Li S., Zhu J., Zhang F., Liang S., Zhang W., Guan Y., Shen D. (2012). A metagenome-wide association study of gut microbiota in type 2 diabetes. Nature.

[58] Lambeth S.M., Carson T., Lowe J., Ramaraj T., Leff J.W., Luo L., Bell C.J., Shah V.O. (2015). Composition, Diversity and Abundance of Gut Microbiome in Prediabetes and Type 2 Diabetes. J. Diabetes Obes..

[59] Frost F., Storck L.J., Kacprowski T., Gartner S., Ruhlemann M., Bang C., Franke A., Volker U., Aghdassi A.A., Steveling A. (2019). A structured weight loss program increases gut microbiota phylogenetic diversity and reduces levels of Collinsella in obese type 2 diabetics: A pilot study. PLoS ONE.

[60] Laserna-Mendieta E.J., Clooney A.G., Carretero-Gomez J.F., Moran C., Sheehan D., Nolan J.A., Hill C., Gahan C.G.M., Joyce S.A., Shanahan F. (2018). Determinants of Reduced Genetic Capacity for Butyrate Synthesis by the Gut Microbiome in Crohn’s Disease and Ulcerative Colitis. J. Crohn’s Colitis.

