# Supplementary material for: Daily Intake of Paraprobiotic Lactobacillus amylovorus CP1563 Improves Pre-Obese Conditions and Affects the Gut Microbial Community in Healthy Pre-Obese Subjects: A Double-Blind, Randomized, Placebo-Controlled Study
Source: Microorganisms. 2020 Feb 22;8(2):304. doi: 10.3390/microorganisms8020304 (PMC7074956; doi:10.3390/microorganisms8020304)
Supplement: Supplementary file 1 [file microorganisms-08-00304-s001.pdf]

Table S1. Abdominal fat areas and anthropometric parameters.

Parameters	Treatment	Values			
		0 weeks	4 weeks	8 weeks	12 weeks
Abdominal total fat area (cm^2)	CP1563-10-HOA	335.0 ± 6.7	339.7 ± 7.1	335.4 ± 7.2	332.0 ± 7.3
	Placebo	340.1 ± 6.3	345.6 ± 6.9	346.1 ± 6.9	349.6 ± 7.0
	p value (vs. placebo)	0.58	0.55	0.29	0.08
Abdominal visceral fat area (cm^2)	CP1563-10-HOA	92.0 ± 3.5	93.1 ± 3.5	89.2 ± 3.2	88.8 ± 3.4
	Placebo	90.8 ± 3.7	90.3 ± 3.4	89.6 ± 3.5	92.4 ± 3.8
	p value (vs. placebo)	0.81	0.55	0.92	0.49
Abdominal subcutaneous fat area (cm^2)	CP1563-10-HOA	242.9 ± 6.5	246.5 ± 6.6	246.2 ± 6.7	243.1 ± 6.6
	Placebo	249.3 ± 6.4	255.3 ± 6.8	256.4 ± 6.9	257.2 ± 6.8
	p value (vs. placebo)	0.48	0.35	0.29	0.14
Body weight (kg)	CP1563-10-HOA	72.5 ± 0.9	72.5 ± 0.9	72.4 ± 0.9	72.3 ± 1.0
	Placebo	74.5 ± 1.0	74.8 ± 1.0	75.0 ± 1.0	75.1 ± 1.0
	p value (vs. placebo)	0.14	0.097	0.06	0.045
BMI (kg/m^2)	CP1563-10-HOA	27.0 ± 0.2	27.1 ± 0.2	27.0 ± 0.2	26.9 ± 0.2
	Placebo	27.1 ± 0.2	27.2 ± 0.2	27.3 ± 0.2	27.3 ± 0.2
	p value (vs. placebo)	0.86	0.58	0.26	0.18
Body fat percentage (%)	CP1563-10-HOA	34.2 ± 0.7	34.4 ± 0.8	34.6 ± 0.8	34.3 ± 0.7
	Placebo	33.9 ± 0.7	34.0 ± 0.8	34.0 ± 0.8	34.1 ± 0.8
	p value (vs. placebo)	0.79	0.67	0.57	0.81
Waist circumference (cm)	CP1563-10-HOA	91.1 ± 0.7	91.4 ± 0.7	91.8 ± 0.6	92.2 ± 0.6
	Placebo	91.4 ± 0.7	91.8 ± 0.7	92.2 ± 0.7	92.6 ± 0.7
	p value (vs. placebo)	0.74	0.61	0.69	0.64

Values are the mean ± SEM. The *p* value was calculated by Student's *t*-test.

Table S2. The α-diversity indices.

	CP1563-10-HOA	Placebo	*p* value (vs. placebo)
**0 weeks**			
Chao1	1089.7 ± 35.8	1081.9 ± 35.5	0.88
Shannon	5.9 ± 0.1	5.8 ± 0.1	0.43
PD whole tree	27.7 ± 0.9	27.0 ± 0.8	0.55
**12 weeks**			
Chao1	1267.7 ± 44.1	1349.0 ± 42.5	0.19
Shannon	6.2 ± 0.1	6.1 ± 0.1	0.85
PD whole tree	31.4 ± 1.0	32.3 ± 0.9	0.49

Values are the mean ± SEM (the CP1563-10-HOA group at 0 weeks: n=83, at 12 weeks: n=82; the placebo group at 0 weeks: n=84, at 12 weeks: n=83). The *p* values were calculated by Student's *t*-test.

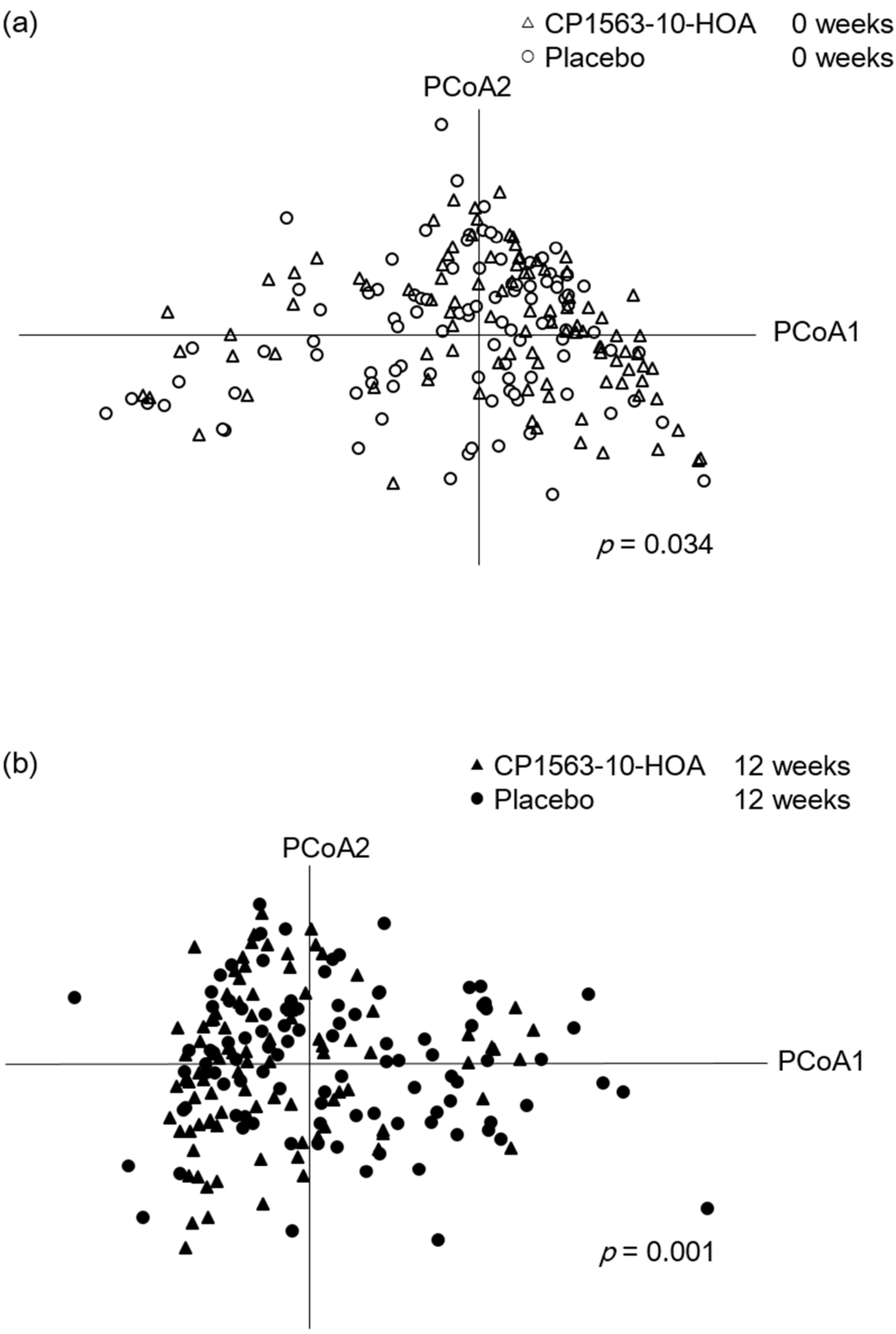

Figure S1. Plots of the placebo and CP1563-10-HOA groups at 0 (a) and 12 (b) weeks were visualized by principal coordinate analysis (PCoA) based on the weighted UniFrac distance (the CP1563-10-HOA group at 0 weeks: n=83, at 12 weeks: n=82; the placebo group at 0 weeks: n=84, at 12 weeks: n=83). The *p* values were calculated by permutational MANOVA based on the weighted UniFrac distance.

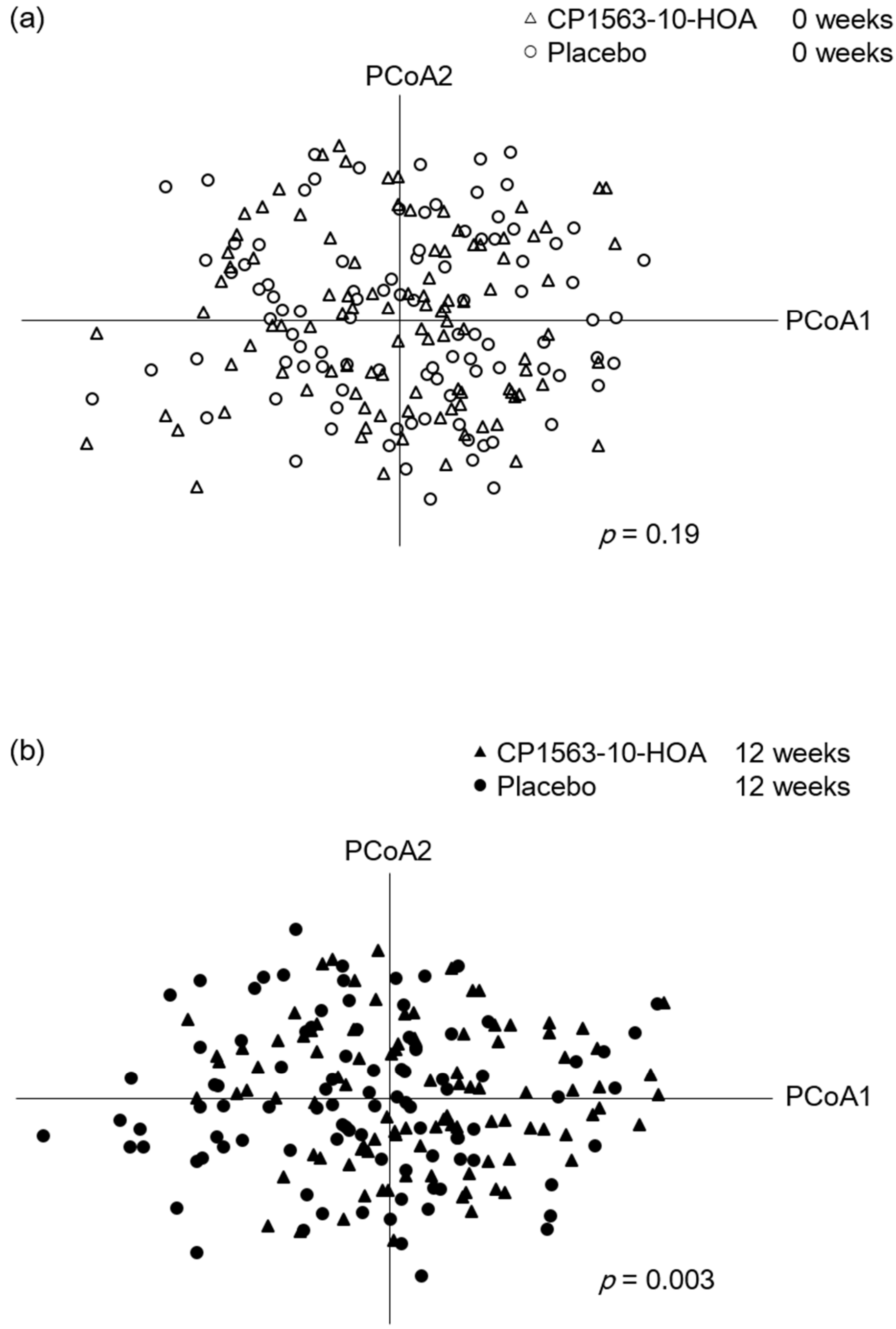

Figure S2. Plots of the placebo and CP1563-10-HOA groups at 0 (a) and 12 (b) weeks were visualized by principal coordinate analysis (PCoA) based on the unweighted UniFrac distance (the CP1563-10-HOA group at 0 weeks: n=83, at 12 weeks: n=82; the placebo group at 0 weeks: n=84, at 12 weeks: n=83). The *p* values were calculated by permutational MANOVA based on the unweighted UniFrac distance.

Table S3. Components of the fecal microbiota at the genus level.

Genus	Composition (%)
Blautia	19.5 ± 0.5
Bifidobacterium	10.3 ± 0.6
Faecalibacterium	10.7 ± 0.5
Coprococcus	9.1 ± 0.3
Lachnospiraceae;g_[Ruminococcus]	6.3 ± 0.3
Lachnospiraceae;g	5.7 ± 0.2
Roseburia	5.8 ± 0.4
Bacteroides	5.5 ± 0.4
Streptococcus	2.6 ± 0.2
Ruminococcus	2.6 ± 0.2
Dorea	2.2 ± 0.1
Erysipelotrichaceae;g_[Eubacterium]	1.3 ± 0.2
Lachnospiraceae; Other	1.3 ± 0.1
Butyricicoccus	1.1 ± 0.1
Collinsella	1.1 ± 0.1

Values are the mean ± SEM (n=332).

Table S4. Summary of the characteristics of subjects and gut microbial composition of this study at week 0 and other studies.

	CP1563-10-HOA	Placebo	Nishijima et al. [1]	Hisada et al. [2]	Oki et al. [3]
BMI (kg/m^2)	27	27	22	25	22
Age (years)	44	42	32	38	52
**Genus (%)**					
Blautia	21.9 ± 1.2	18.4 ± 1.0	16.7	14.8	4.4
Bifidobacterium	8.0 ± 1.1	11.8 ± 1.4	17.9	4.6	2.8
Faecalibacterium	10.9 ± 1.0	9.4 ± 0.7	5.8	5.3	7.5
Coprococcus	9.5 ± 0.8	9.9 ± 0.8	1.4	1.4	2.2
Lachnospiraceae;g_[Ruminococcus]	6.5 ± 0.6	7.0 ± 0.7	-	-	0.4
Lachnospiraceae;g	4.7 ± 0.4	6.5 ± 0.5	-	-	0.4
Roseburia	5.7 ± 0.7	6.2 ± 0.8	2.2	1	1.2
Bacteroides	5.8 ± 0.6	3.3 ± 0.5	11.4	17.7	28.2
Streptococcus	3.0 ± 0.5	2.4 ± 0.5	2.2	2.5	1.1
Ruminococcus	2.3 ± 0.3	2.8 ± 0.4	5.6	7.1	3.3
Dorea	2.3 ± 0.3	2.3 ± 0.2	2.1	1.4	0.7
Erysipelotrichaceae;g_[Eubacterium]	1.6 ± 0.4	1.1 ± 0.3	-	-	0.6
Lachnospiraceae; Other	1.2 ± 0.2	1.3 ± 0.1	-	-	6.5
Butyricicoccus	1.1 ± 0.1	1.3 ± 0.1	-	-	0.01
Collinsella	1.0 ± 0.1	1.3 ± 0.2	3.0	2.7	0.6

Values of characteristics of subjects are the mean. Values of gut microbial composition are the mean ± SEM (this study) or the mean (other studies).

References

1. Nishijima, S.; Suda, W.; Oshima, K.; Kim, S.W.; Hirose, Y.; Morita, H.; Hattori, M. The gut microbiome of healthy Japanese and its microbial and functional uniqueness. *DNA Res.* **2016**, *23*, 125-133, doi:10.1093/dnares/dsw002.

2. Hisada, T.; Endoh, K.; Kuriki, K. Inter- and intra-individual variations in seasonal and daily stabilities of the human gut microbiota in Japanese. *Arch. Microbiol.* **2015**, *197*, 919-934, doi:10.1007/s00203-015-1125-0.

3. Oki, K.; Toyama, M.; Banno, T.; Chonan, O.; Benno, Y.; Watanabe, K. Comprehensive analysis of the fecal microbiota of healthy Japanese adults reveals a new bacterial lineage associated with a phenotype characterized by a high frequency of bowel movements and a lean body type. *BMC Microbiol.* **2016**, *16*, 284, doi:10.1186/s12866-016-0898-x.
